# Identification of ferroptosis-related genes for overall survival prediction in hepatocellular carcinoma

**DOI:** 10.1038/s41598-022-14554-7

**Published:** 2022-06-15

**Authors:** Lianxiang Luo, Xinyue Yao, Jing Xiang, Fangfang Huang, Hui Luo

**Affiliations:** 1 The Marine Biomedical Research Institute, Guangdong Medical University, Zhanjiang, 524023 Guangdong China; 2 Southern Marine Science and Engineering Guangdong Laboratory (Zhanjiang), Zhanjiang, 524023 Guangdong China; 3 The Marine Biomedical Research Institute of Guangdong Zhanjiang, Zhanjiang, 524023 Guangdong China; 4 The First Clinical College, Guangdong Medical University, Zhanjiang, 524023 Guangdong China; 5 Graduate School, Guangdong Medical University, Zhanjiang, 524023 Guangdong China

**Keywords:** Cancer, Computational biology and bioinformatics, Genetics

## Abstract

Ferroptosis is a novel type of cell death depending on iron and is strongly related to the development of tumors. Hepatocellular carcinoma (HCC) is a malignancy with high incidence. Despite some reports demonstrating the relation between ferroptosis-related genes and HCC, more details have not been excavated. In the present study, we collected and analyzed HCC patients' datasets from the TCGA-LIHC project and ICGC portal, respectively. Through the bioinformatic methods, we screened 126 differentially expressed genes. Then a prognostic model was established with four genes (GPX2, MT3, PRDX1, and SRXN1). PRDX1 is the hub gene of the prognosis model and has a high expression in hepatocellular carcinoma tumor tissue and cell lines. We further found that silencing PRDX1 increased the accumulation of ferrous ions and lipid peroxidation accumulation in HEPG2 cells and promoted ferroptosis in hepatocellular carcinoma. In conclusion, the study demonstrated the four-gene signature can be used to predict HCC prognosis. It also revealed the potential function of the ferroptosis-related gene PRDX1 in HCC, which can be a biomarker of the prediction for HCC outcome.

## Introduction

Hepatocellular carcinoma (HCC) is one of the most common heterogeneous malignancies, which is the sixth most widespread neoplasm and the fourth leading cause of cancer death^1^. The incidence and mortality of liver cancer are gradually increasing, and HCC is accounting for ~ 90% of primary liver cancers^1^. Concerning the complex pathogenesis of HCC, some reports about the etiology and development of HCC have speculated that it is associated with cirrhosis, viral hepatitis, specific chemical carcinogens, and abnormal regulation of hormones^2^. However, early diagnosis and treatment of HCC remain an issue, especially in the low-level medical developing countries^3^. Although the diagnostic and therapeutic techniques for HCC are developing rapidly^4^, the prognosis of HCC is very poor, approximately 70% of HCC relapse within 5 years after receiving resection or ablation^5^. Currently, due to the not obvious early clinical symptoms, most of the patients were firstly diagnosed in the middle or late stages so that they miss the optional treatment period. Consequently, it needs more effort to identify early diagnostic biomarkers of HCC, and their utilization is beneficial for timely and effective treatment for patients.

Ferroptosis is an iron-independent form of cell death, the features and mechanisms are different from apoptosis, necrosis, and autophagy, which is characterized by the accumulation of lipid reactive oxygen species (ROS)^6^. The cystine/glutamate antiporter (system Xc^−^) and Glutathione peroxidase-4 (GPX4) in the typical glutathione pathway are two vital regulatory points of ferroptosis mechanisms^7^. The system Xc^−^ can regulate the exchange of intracellular glutamate and extracellular cystine^8^. And GPX4, as a unique member of selenium-dependent glutathione peroxidase distributed within mammals, plays a pivotal role in suppressing the generation of lipid ROS during ferroptotic cell death^9^. Interestingly, cysteine impedes the biosynthesis of glutathione (GSH), which is a substrate of GPX4. Therefore, the interaction of the system Xc^−^ and GPX4 induces the accumulation of ROS and ferroptosis^6^. Sorafenib, as the standard first-line drug against advanced HCC, could inhibit system Xc^−^ and induce the ferroptosis to exert its cytotoxic effects^10–12^. Some previous studies have also reported the importance of ferroptosis for the treatment and prognosis of liver cancer^13–16^, but the hub ferroptosis-related regulators and potential regulatory mechanism of ferroptosis during the occurrence and progression of HCC have not been investigated in detail.

In this study, we systematically analyzed some ferroptosis-related genes in HCC based on the TCGA (The Cancer Genome Atlas, https://portal.gdc.cancer.gov/repository) datasets and try to clarify how they affect the pathogenesis and prognosis of HCC. We hope that the biomarkers will be helpful for the diagnosis, treatment, and prognosis of HCC.

## Results

### Identification of differentially expressed FRGs

**Figure 1:**
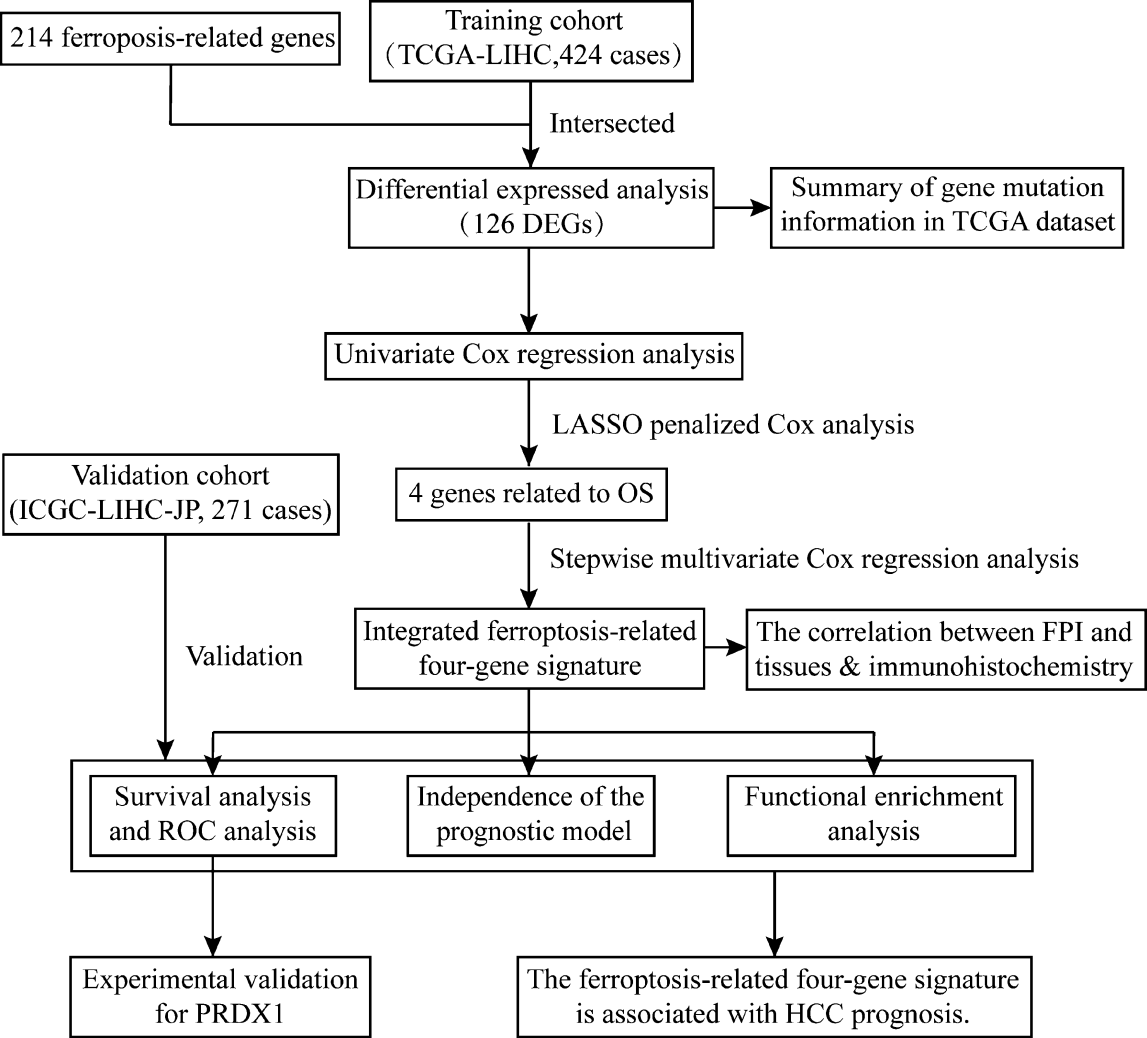
A flow chart for overall study design.

**Figure 2:**
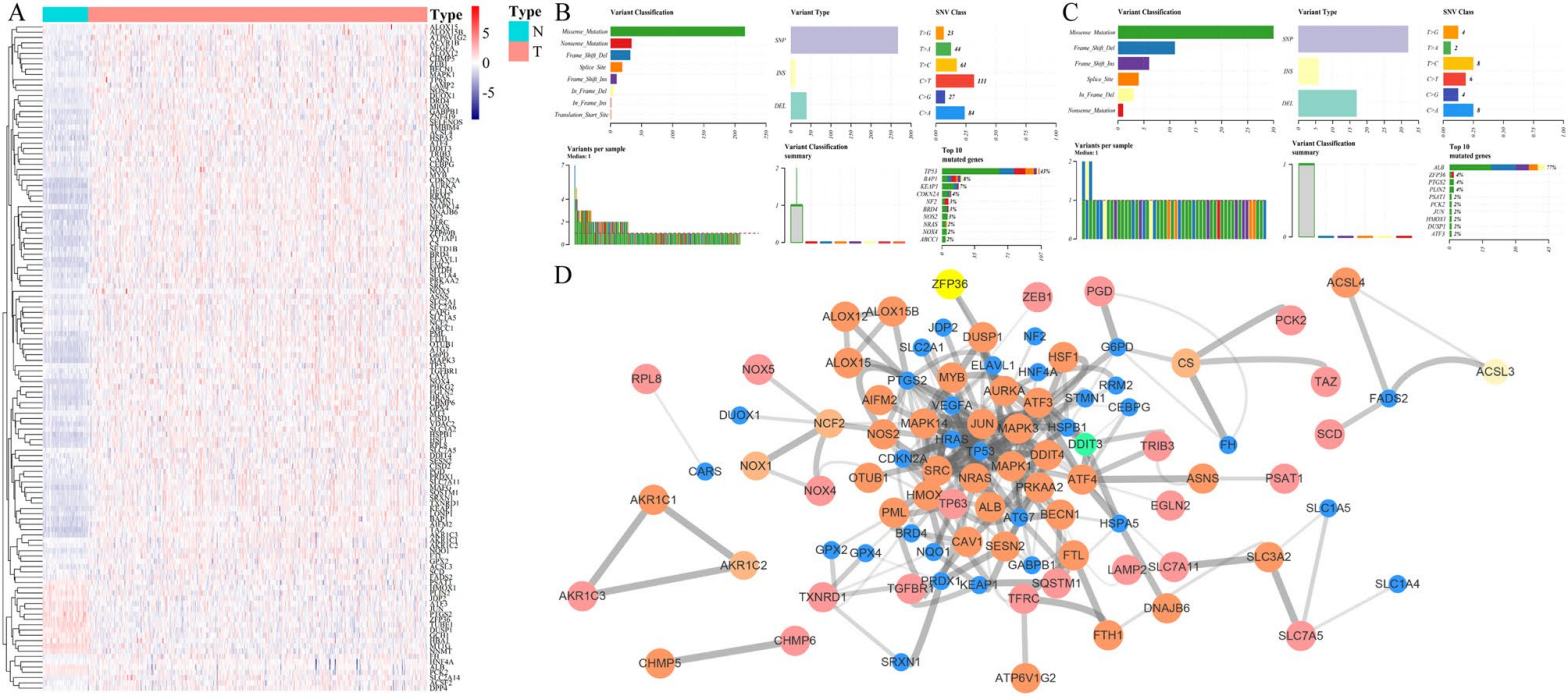
Upregulated and downregulated differentially expressed genes in hepatocellular carcinoma patients. (**A**) A heatmap showing the expressions of the 126 differentially expressed genes in the normal tissues and tumors of The Cancer Genome Atlas (TCGA) cohort. (**B,C**) Summary of gene mutation information in the up-regulated genes (**B**) and down-regulated genes (**C**) in the TCGA dataset. (**D**) The PPI network indicated the interactions among the differentially expressed genes.

A flow chart was performed to completely describe our study (Fig. 1). The training cohort was obtained from the TCGA-LIHC project, which contained 374 tumor samples and 50 normal samples. Meanwhile, we acquired 214 ferroptosis-related genes (FRGs) from the FerrDb database (http://www.zhounan.org/ferrdb) that included 98 drivers, 94 suppressors, and 101 makers. Intersecting two datasets to get a list of 200 ferroptosis-related genes with their expression profile in the TCGA-LIHC cohort. The “limma” R package was used to analyze the expression pattern of FRGs. The analysis identified 126 differentially expressed FRGs, including 110 high expressed genes and 16 low expressed genes (Fig. 2A). Then, we tried to describe the overall condition of the simple nucleotide variation for 126 differentially expressed genes (DEGs). And we found that the variant classification and variant type between up-regulated genes and down-regulated genes have the same distributions (Fig. 2B,C). To investigate the correlation of all the DEGs, make an analysis of these DEGs by STRING website (https://string-db.org/). The result, a PPI network, was visualized by Cytoscape software (version 3.8.2), and the orange means high expression but the blue means low expression (Fig. 2D).

### Establishment and assessment of prognostic model

**Figure 3:**
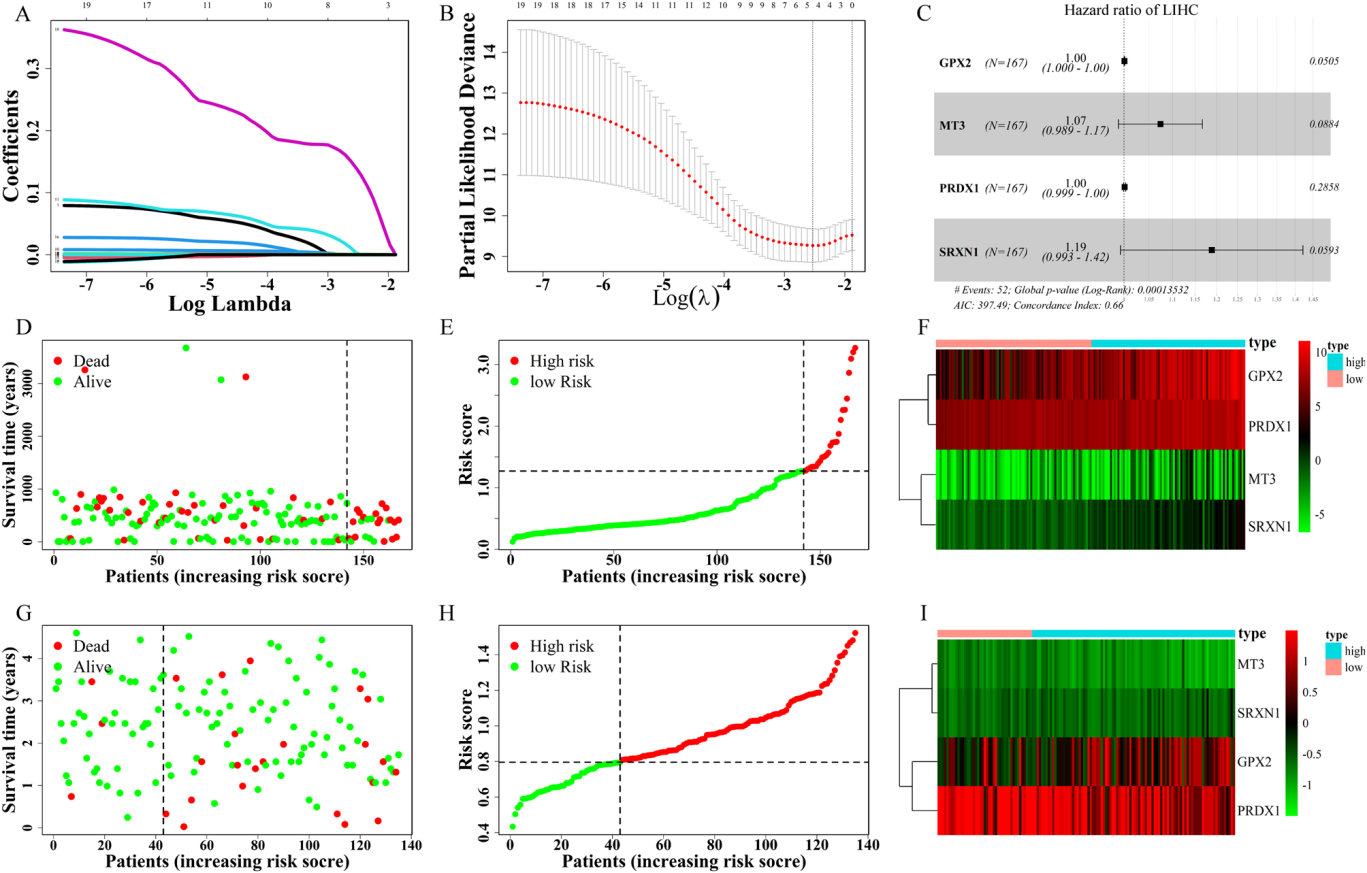
The process of building the prognostic gene signature and a signature-based risk score is a promising marker in TCGA and International Cancer Genome Consortium (ICGC) cohorts. (**A**) Distribution of LASSO coefficients of the 19 ferroptosis-related potential prognostic genes in the training cohort. (**B**) The generated coefficient distribution plots for the logarithmic (lambda) sequence for the selection of the best parameter (lambda). (**C**) Forest plot showing the ferroptosis-related gene associated with the survival of patients with hepatocellular carcinoma. (**D–I**) The left represents the survival status of patients in the training (**D**) and validation (**G**) cohorts; the center represents the distribution of the risk score in the training (**E**) and validation (**H**) cohorts; the right represents the expression pattern of the prognostic signature genes in the classifiers of the high- and low-risk groups in the training (**F**) and validation (**I**) cohorts.

**Figure 4:**
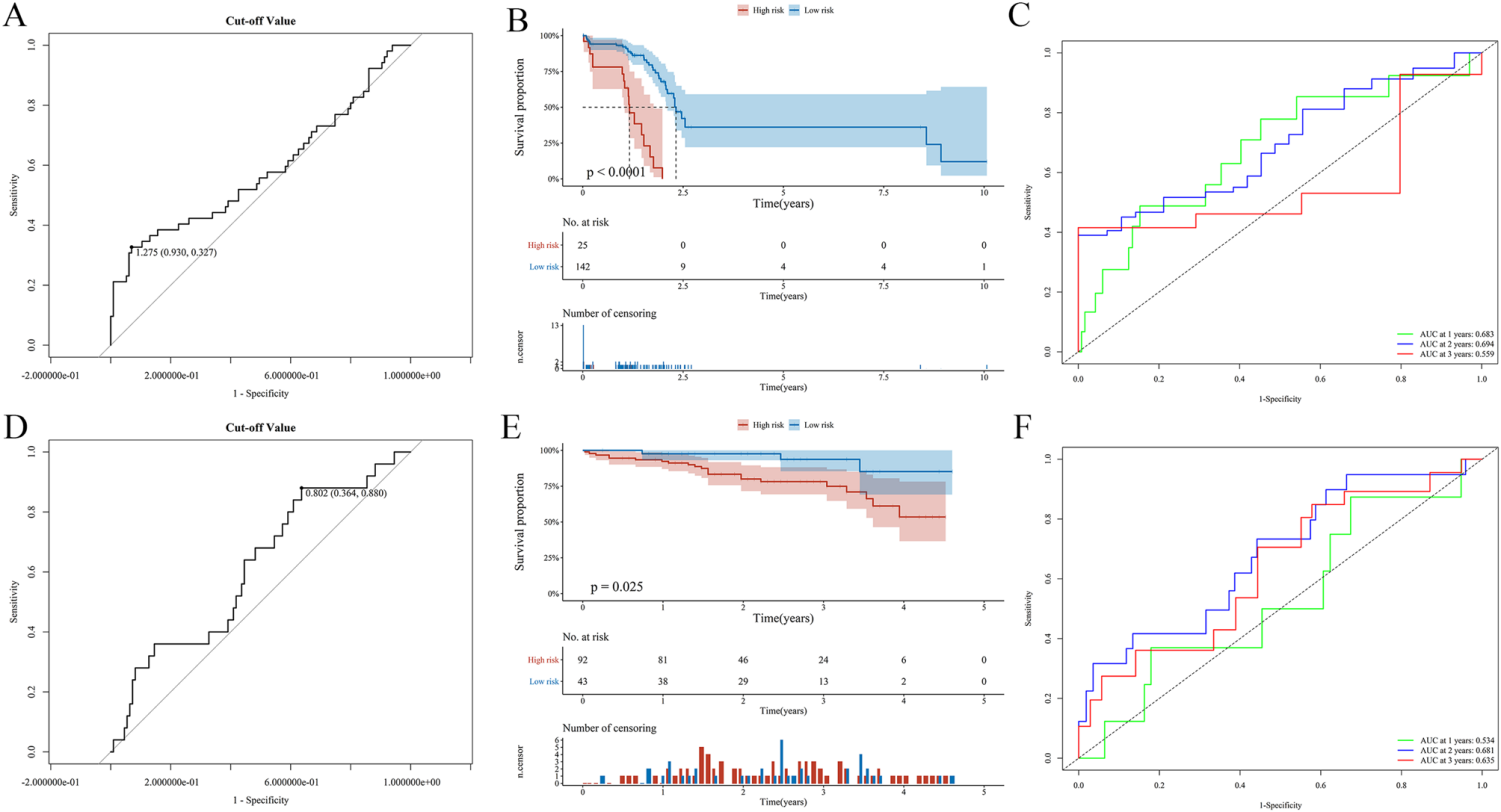
Kaplan–Meier analysis and time-dependent ROC analysis in training and validation cohorts. (**A,D**) The cut-off value in the TCGA (**A**) and ICGC (**D**) cohorts. (**B,E**) Kaplan–Meier overall survival (OS) curves for patients in the high-risk group and low-risk group in the TCGA (**B**) and ICGC (**E**) cohorts. (**C,F**) ROC curves showed the predictive efficiency of the risk signature for patients in the TCGA (**C**) and ICGC (**F**) cohorts on the survival rate.

To understand what role FRGs play in the HCC more deeply, we applied univariate Cox regression analysis and received 19 prognostic genes (Table 1). The LASSO (Least Absolute Shrinkage and Selection Operator) regression analysis was used to get the genes that are obviously related to the overall survival of HCC (Fig. 3A), and cross-validation for the model of selected genes is necessary (Fig. 3B). For a stable prognosis-related gene signature, the 19 genes related to overall survival (OS) were processed by stepwise multivariate Cox regression analysis after the LASSO approach. Following that, we finally built a four-gene signature for overall survival in HCC, and the four genes are GPX2, MT3, PRDX1, and SRXN1 (Fig. 3C). According to the multivariate Cox regression coefficients, the risk score of each sample in the training cohort was calculated. To assure the accurate separating standard of the risk score group, we painted an ROC curve to search cut-off value based on the risk score, and the cut-off value we got is 1.275 (Fig. 4A). The 167 HCC patients in the training dataset were divided into two groups, high- (n = 25) and low-risk group (n = 142), based on the cut-off value. Comparing two groups with their survival status and the four genes expression, the high-risk group is obviously associated with a comparative high death rate and with higher gene expression (Fig. 3D–F). Through the same process, the HCC patients in validation cohort were divided into high- (n = 92) and low-risk group (n = 43) by cut-off value (cut-off value = 0.802) (Fig. 4D). And the results that the correlation in risk score and survival status even genes expression are similar to the training set (Fig. 3G–I).

**Table 1:** Univariate Cox Regression Analysis of DEGs in The Cancer Genome Atlas.

Gene	HR	95% CI	*P* value
ABCC1	1.081	1.008–1.159	0.029
AKR1C1	1.003	1.001–1.005	0.001
AKR1C2	1.005	1.001–1.009	0.017
AKR1C3	1.005	1.001–1.008	0.015
EMC2	1.075	1.006–1.147	0.032
FTH1	1.002	1.000–1.003	0.037
FTL	1.000	1.000–1.000	0.010
G6PD	1.009	1.003–1.006	0.006
GPX2	1.001	1.001–1.002	< 0.001
HMOX1	1.013	1.004–1.022	0.005
MT3	1.110	1.030–1.196	0.006
NNMT	1.001	1.001–1.002	< 0.001
NQO1	1.003	1.001–1.005	0.007
PGD	1.007	1.002–1.012	0.005
PRDX1	1.003	1.002–1.005	< 0.001
SLC2A1	1.047	1.006–1.090	0.024
SQSTM1	1.006	1.003–1.009	< 0.001
SRXN1	1.353	1.187–1.542	< 0.001
TXNRD1	1.018	1.010–1.026	< 0.001

Abbreviations: HR, hazard ratio; CI, confidence interval.

Next, we applied Kaplan–Meier analysis to perform the difference of survival status between the high-risk group and low-risk group in the training cohort (Fig. 4B). The Kaplan–Meier curve presents the significant difference between the high- and low-risk group (*P* < 0.0001). For better convincing, we painted the ROC curve to validate the result of the Kaplan–Meier curve, and a higher AUC (Area Under Curve) means better performance. The AUCs of OS corresponding to 1, 2, and 3 years in the training set were 0.682, 0.694, and 0.539, respectively (Fig. 4C). The two plots were also validated in the testing cohort, and we got the same result that the Kaplan–Meier curve performed a differentially expressed OS between two groups (*P* = 0.025) (Fig. 4E), AUCs were 0.534, 0.681, and 0.635 respectively contacting to the OS of 1, 2 and 3 years in the validation set (Fig. 4F). The credible AUCs can prove the high-risk group with poor prognosis presented by the Kaplan–Meier curve in HCC patients. Besides, we also analyzed the four prognostic genes with the Kaplan–Meier approach in the training cohort and validation dataset, respectively (Fig. S1A–H). And we noticed that the expression level of PRDX1 is obviously related to overall survival (*P* = 0.031; *P* = 0.023). However, the expression of the four genes in the normal group, tumor group, and risk groups did not present differences (Fig. S1I, J). And then we used the STRING website to determine the key of the four genes are PRDX1 and SRXN1 (Fig. S2A–C). Through the exploration of the GEPIA website, we checked the expression level of the two genes and plotted their survival curves based on the TPM value of the expression level (Fig. S2D–G).

**Figure 5:**
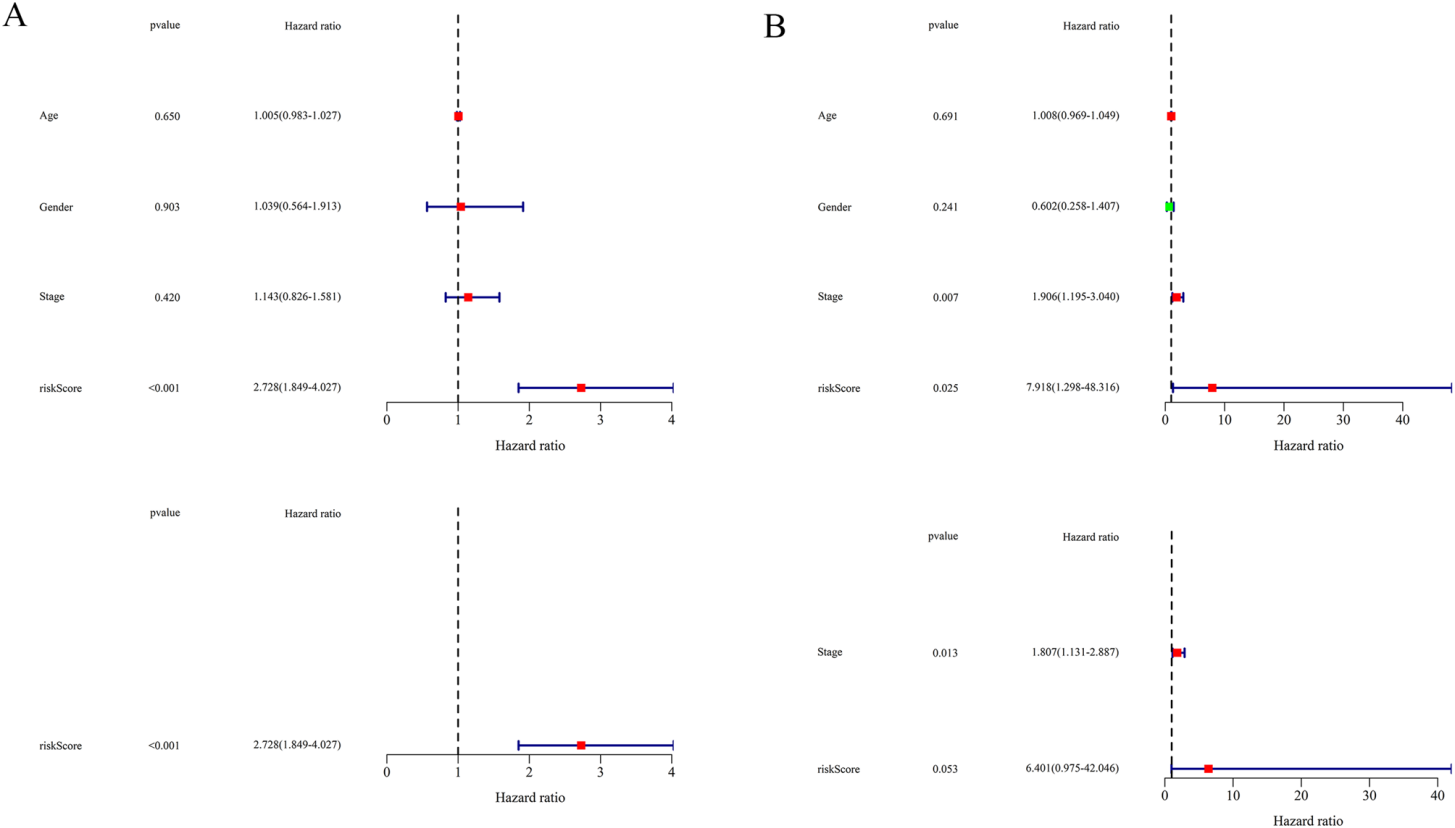
Independent prognostic analysis of risk scores and clinical parameters. (**A,B**) The univariate and multivariate Cox regression analyses of the associations between the risk scores and clinical parameters and the OS of patients in the TCGA (**A**) and ICGC (**B**) cohorts.

For the prognostic model, univariate Cox regression analysis and multivariate Cox regression analysis were applied to assess the clinical characteristics whether are the independent predictively prognostic factors or not. As shown in Fig. 5, with four clinical parameters (Age, Gender, Stage, and risk score), it is suggested that risk score is the most significant clinical feature related to survival in training and testing cohorts (*P* < 0.05), but the p-value of risk score processed by multivariate Cox regression analysis in ICGC (International Cancer Genome Consortium, https://dcc.icgc.org/projects/LIRI-JP) dataset (*P* = 0.053) is a few more than 0.05. Hence, we considered that risk score can be an independent prognostic predictor with high probability.

### Association between FRGs and immune

**Figure 6:**
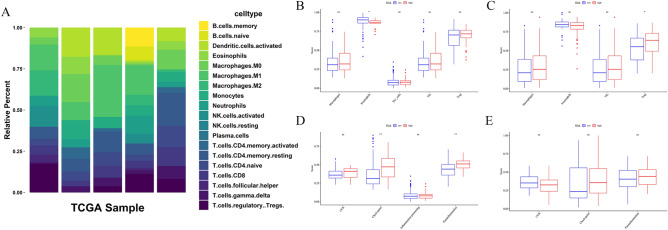
Tumor-infiltrating immune cell (TIC) distribution map and the single-sample gene set enrichment analysis (ssGSEA) scores between different risk groups in the TCGA and ICGC cohorts. (**A**) The bar graph shows the relative content distribution of 19 TICs of hepatocellular carcinoma in the training cohort. (**B**) Boxplots of the correlations between the scores of 5 immune cells and risk groups in TCGA. (**C**) Boxplots of the correlations between the scores of 4 immune cells and risk groups in ICGC. (**D**) Boxplots of the correlations between 4 immune-related functions and risk groups in TCGA. (**E**) Boxplots of the correlations between the scores of 3 immune cells and risk groups in ICGC. Adjusted p-values are shown as: ns, not significant (*P < 0.05; **P < 0.01).

To clearly evaluate the proportion of 19 tumor-infiltrating immune cells in each sample, CIBERSORT analysis was applied to HCC samples which *P* value < 0.05 in the training dataset (Fig. 6A). Besides, we explored the correlation between risk score and immune status including immune cells and immune functions. Single-sample Gene Set Enrichment Analysis (ssGSEA) was applied to assess the abundance level of immune cells and immune functions in two cohorts (Fig. 6B–E), most enrichment scores expressed highly in the high-risk group. Figure 6B and Fig. 6C presented the different enrichment scores of 5 immune cells and 4 immune functions between high- and low-risk groups in the training dataset. And the enrichment score of neutrophils in 5 immune cells was significantly different in the two risk groups (*P* < 0.05), the enrichment scores of check-point and Para inflammation in 4 immune functions indicated significant difference (*P* < 0.01). As shown in Fig. 6D and E**,** they presented different enrichment scores of 4 immune cells and 3 immune functions between high- and low-risk groups in the validation dataset. However, the results were different from those in the training cohort. The enrichment score of Treg cells in 4 immune cells was significantly different in two risk groups (*P* < 0.05), but there were not obviously different enrichment scores about immune functions between high- and low-risk groups in the testing cohort.

### Gene ontology and Kyoto Encyclopedia of Genes and Genomes pathway enrichment analyses

**Figure 7:**
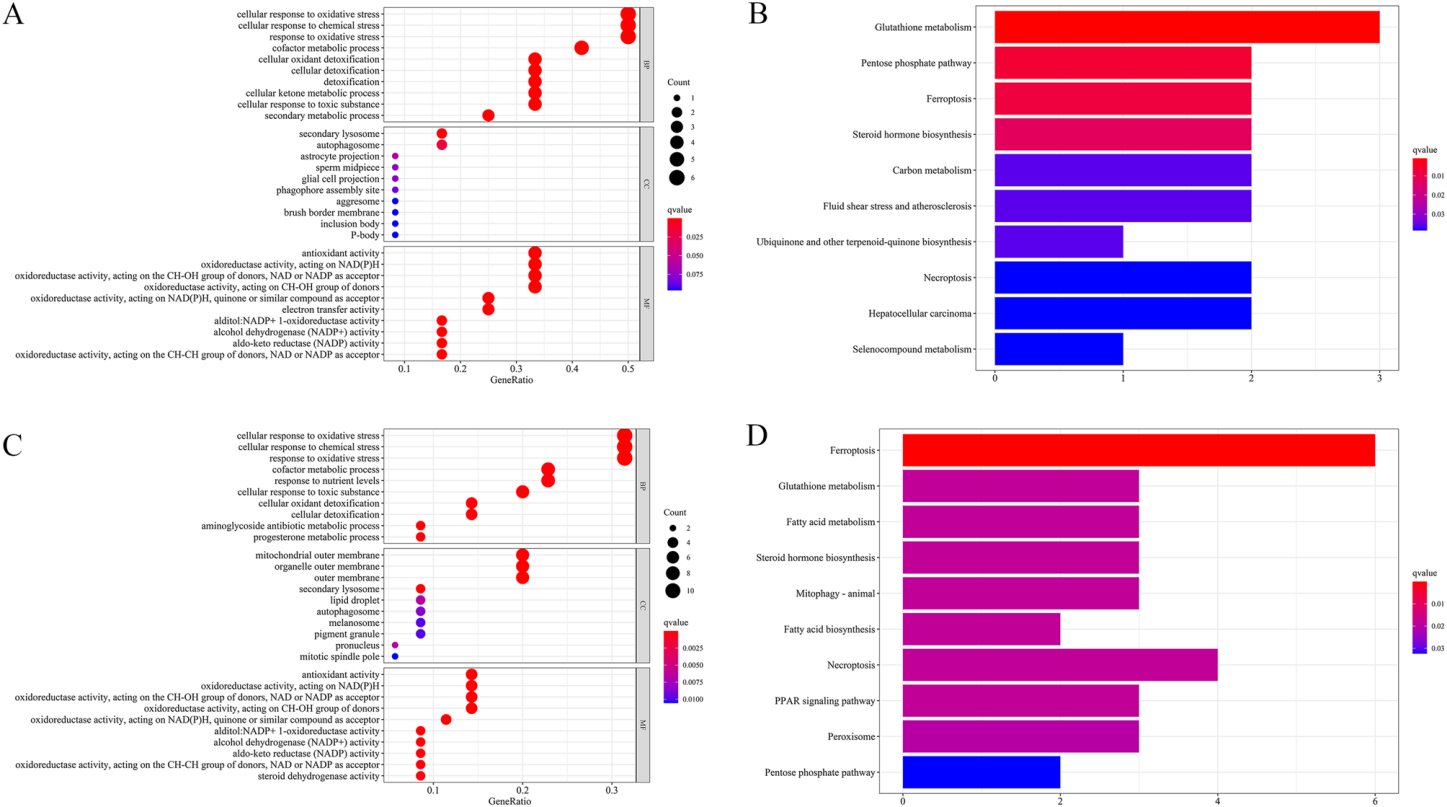
Gene Ontology (GO) and Kyoto Encyclopedia of Genes (KEGG) enrichment analysis in training and validation cohorts. (**A,C**) GO analysis based on prognostic ferroptosis-related genes in TCGA (**A**) and ICGC (**C**) cohorts. (**B,D**) KEGG pathway analysis based on prognostic ferroptosis-related genes in TCGA (**B**) and ICGC (**D**) cohorts.

Subsequently, we applied functional enrichment analyses to the DEGs in high- and low-risk groups in the training cohort. As observed, the result of functional annotation encompassing biological processes (BP), cellular components (CC), and molecular functions (MF) by Gene Ontology (GO) analysis performed some ferroptosis-related function, such as cellular response to oxidative stress in biological processes, antioxidant activity, oxidoreductase activity, acting on NAD(P)H in molecular functions and so on (Fig. 7A). In addition, the ferroptosis-related DEGs were dealt with Kyoto Encyclopedia of Genes and Genomes (KEGG) pathway enrichment analysis (Fig. 7B). As expected, the pathway was significantly enriched on the ferroptosis pathway. To guarantee the result more accurately, ICGC cohort, a validation dataset was accepted with GO and KEGG pathway enrichment analyses (Fig. 7C,D), too. Delightfully, the functions and pathways of enrichment in the validation dataset were as same as the training dataset, especially the enriched pathways found by KEGG analysis that the first enrichment pathway was ferroptosis.

### The correlation between FPI and tissues and immunohistochemistry for validation

**Figure 8:**
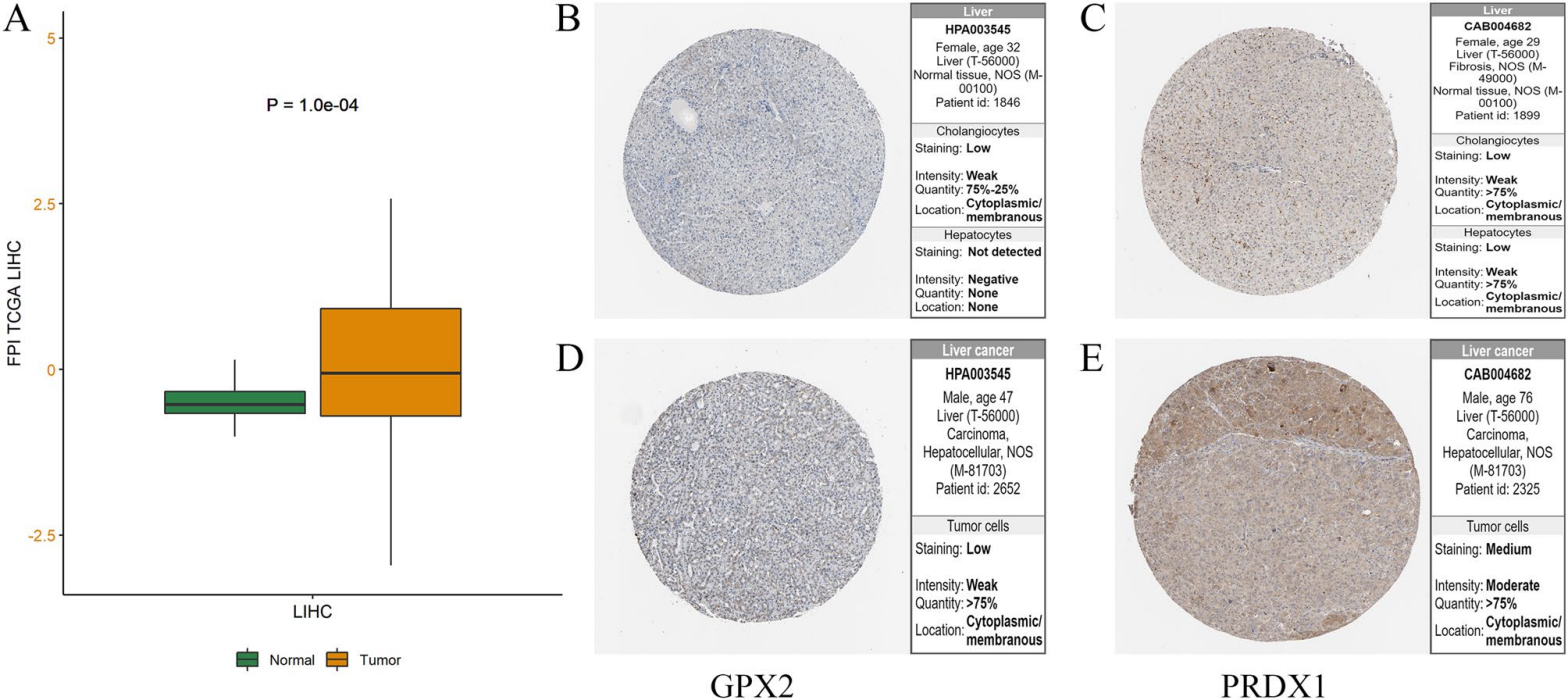
The relations between FPI and tissues and different protein expressions in four genes were verified in human tissue samples. (**A**) The different FPIs between tumor and normal samples among HCC. (**B–E**) Human Protein Atlas (HPA) immunohistochemistry using anti-GPX2 and anti-PRDX1 antibodies. Normal liver (**B,C**) vs. tumor tissues (**D,E**).

To understand the influence on prognosis and therapy ferroptosis brought in HCC, ferroptosis potential index (FPI) quantizing the expression level of ferroptosis was established to reveal the ferroptotic functions and different expressions between normal and tumor tissues in HCC patients from the TCGA-LIHC cohort. As shown in Fig. 8A, the expression level of FPI has an obvious difference (*P* = 1.0e−04) in normal and tumor groups.

Finally, we found out the figures of immunohistochemistry (IHC) about the expression of encoding proteins by four-gene signature from The Human Protein Atlas (HPA) database. Obviously, for the expression level, PRDX1 was strongly expressed in normal tissues, but GPX2 was moderately expressed in tumor tissues (Fig. 8B–E). Regrettably, there were no IHC plots of MT3 and SRXN1 that can be completely compared with on the website.

### Silencing PRDX1 induces ferroptosis in hepatocellular carcinoma cells

**Figure 9:**
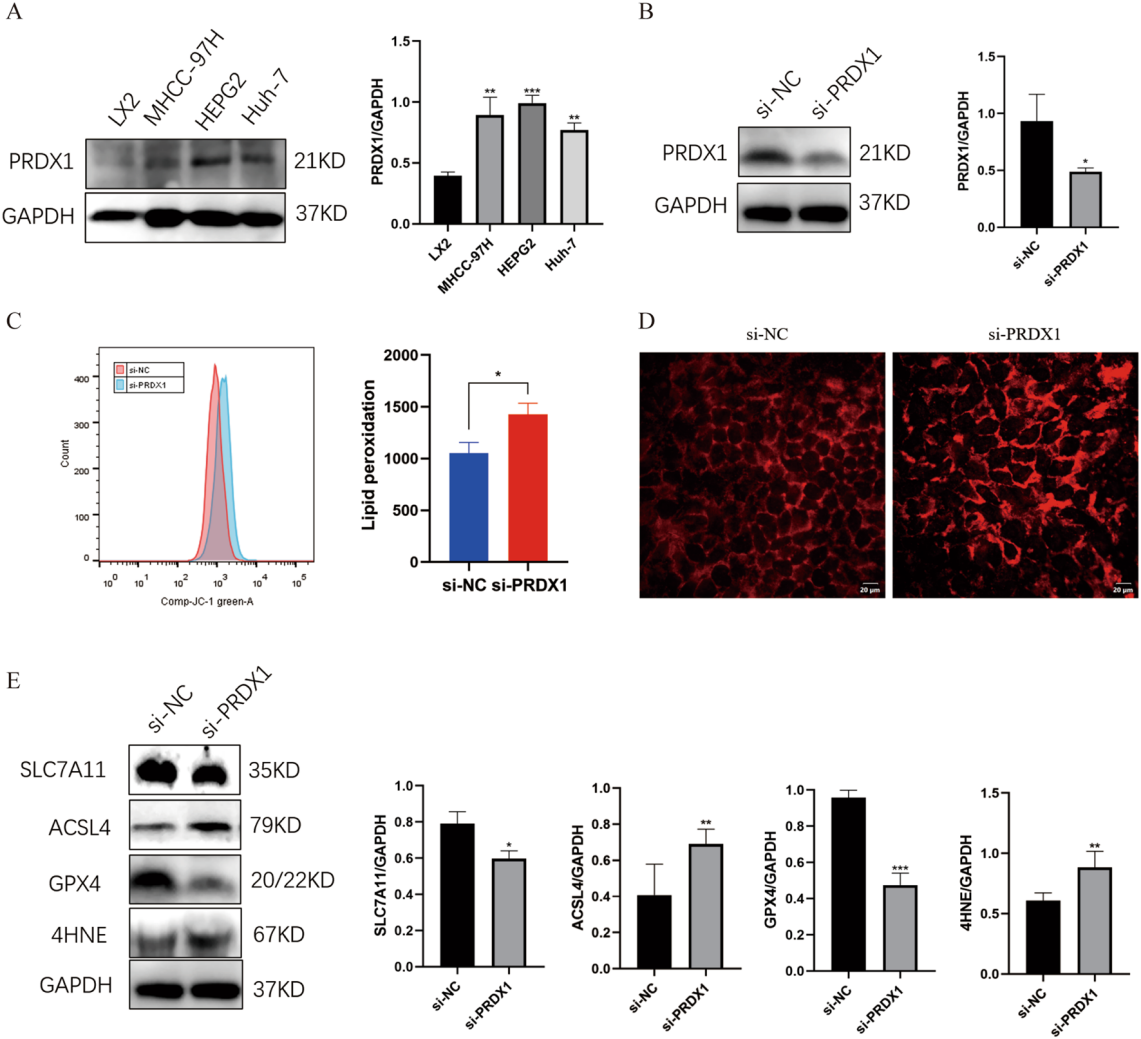
PRDX1 expression is upregulated in hepatocellular carcinoma cell lines and silencing PRDX1 promotes ferroptosis in hepatocellular carcinoma cells. (**A**) The PRDX1 expression levels in hepatocellular carcinoma cell lines using Western Blot. (**B**) PRDX1 was successfully knocked down by siRNA. (**C**) HEPG2 cells had elevated lipid peroxidation levels after silencing PRDX1. (**D**) HEPG2 cells had higher levels of Fe2+ after silencing PRDX1. (**E**) western blot showed decreased expression levels of SLC7A11 and GPX4 and increased expression levels of 4HNE and ACSL4 in HEPG2 cells after silencing PRDX1.

To determine the clinical relevance of PRDX1 expression levels, we examined PRDX1 expression in a normal liver cell (LX2) and three hepatocellular carcinoma cell lines (MHCC-97H, HEPG2, and Huh-7). The results showed that PRDX1 protein levels were expressed in MHCC-97H, HEPG2, and Huh-7 cells (Fig. 9A), and notably, its expression was highest in HEPG2 cells. Therefore, HEPG2 cells were used for the follow-up analysis. To reveal the biological function of PRDX1 in HEPG2 cells, we transfected HEPG2 cells with siRNA to silence PRDX1 expression. Western blot results showed that PRDX1 protein expression was significantly down-regulated in HEPG2 cells after transfection with si -PRDX1, indicating that PRDX1 gene was successfully knocked down (Fig. 9B).

Similarly, to further investigate the role of PRDX1 expression in ferroptosis, we examined the expression of key indicators associated with ferroptosis (Fe2+, lipid peroxidation, and ferroptosis -associated proteins) in HEPG2 cells with knockdown of PRDX1. First, lipid peroxidation plays a key role in the development of ferroptosis, we next examined the effect of PRDX1 on intracellular lipid peroxidation levels in HEPG2 cells and showed that inhibition of PRDX1 expression increased intracellular lipid peroxidation levels in HEPG2 cells (Fig. 9C). In addition, we examined the effect of PRDX1 on the change of intracellular Fe2+ levels and found that PRDX1 knockdown increased intracellular Fe2+ levels in HEPG2 cells (Fig. 9D). Meanwhile, western blot results showed that the expression of SLC7A11 and GPX4 decreased and the expression of 4HNE and ACSL4 increased in HEPG2 cells after PRDX1 knockdown (Fig. 9E). Thus, our results suggest that silencing PRDX1 promotes ferroptosis in hepatocellular carcinoma cells.

## Discussion

By the increasingly developed anticancer therapy of selective induction of cancer cell death, ferroptosis was received with great concern because of its special modality of cell death. Several reports have indicated that ferroptosis plays a crucial role in the process of tumorigenesis^17,18^ and it provides a new perspective on cancer treatment and may develop new strategies for the treatment of liver cancer^19^. Hepatocellular carcinoma has a strong relation with metabolic disorders^20^. ROS has been proved to be chemically reactive metabolites containing oxygen that decrease in liver cancer^21^. Lipid ROS accumulating too much by exogenous drugs and iron-metabolism dysregulation both are the keys in ferroptosis origin and development^6,22^. There are several previous studies had explored the relationship between ferroptosis-related genes and HCC prognosis, and received a few genes related to HCC prognosis^23–25^. However, the mechanism that how ferroptosis influences tumor cell death in HCC patients and which kind of role the predictors for prognosis play is not demonstrated completely until now. Hence, our study constructed a prognostic model with four ferroptosis-related genes through bioinformatic methods, which was proved to be beneficial for early diagnosing HCC patients. Besides, we performed some experiments to validate the role PRDX1 played between HCC and ferroptosis.

In the present study, we constructed a risk model based on prognostic genes, none of the clinical parameters were proved to be an independent prognostic factor except risk score. Then we conducted a series of enrichment analyses on functions and pathways. Based on the close association between ferroptosis and oxidative stress^26^, we observed the functions of cellular response to oxidative stress, oxidoreductase activity, acting on NAD(P)H, and so on were related with ferroptosis in two datasets. Interestingly, the depletion of NADPH will promote lipid ROS accumulation by reducing the GSH which metabolism can directly influence ferroptosis sensitivity^26,27^. Moreover, the KEGG pathway analysis indicated that glutathione metabolism and ferroptosis pathways were enriched, which suggested that all DEGs were strongly linked with ferroptosis. Besides, we also used ssGSEA to construct FPI to characterize ferroptosis based on genes expression patterns and the FPI is higher in tumor tissues. And FPI was put forward to model ferroptosis level which can predict poor prognosis with a high score in many cancer types^28^.

The prognostic model contained four genes-GPX2, MT3, PRDX1, and SRXN1. Glutathione peroxidase 2 (GPX2), a member of the antioxidant enzyme GPX family, overexpression can induce poor prognosis of hepatocellular carcinoma patients^29^. In our study, the expression of GPX2 was high in the high-risk group which has a poor prognosis. Metallothionein III (MT3) overexpression contributes to carcinogenesis and poor prognosis of several other types of cancer patients but not clearly for HCC patients^30^. And with the downregulation of MT3 often accompanying the methylation, there was speculation that MT3 may suppress the tumor via promoting hypermethylation^31^. Up-regulated peroxiredoxin 1 (PRDX1) often acts as an oncogene in many types of cancer but remains controversial^32^. However, it was confirmed that overexpressed PRDX1 was closely related to the poor prognosis of HCC patients^33^. Sulfiredoxin-1 (SRXN1) was revealed as a pro-tumorigenic in HCC by regulating ROS signaling^34^. In addition, SRXN1 is involved in oxidoreductase activity^35^, its overexpression may play a crucial role in the tumorigenesis and progression of HCC^36^. Nevertheless, there are not so many reports about the relationship between these genes and ferroptosis. We can only know that the expression of GPX2, MT3, and SRXN1 was upregulated during ferroptosis induced by erastin or RSL3, and they may promote ferroptosis^7^. Besides, PRDX1 is necessary to ferroptosis-related lipid peroxidation, and it also may promote ferroptosis because ferroptosis-related suppressors can block enhanced lipid peroxidation and recover cell viability without PRDX1^37^. After the survival analysis for each prognostic gene based on its expression level, PRDX1 with high expression is significantly related to low overall survival.

As a member of the PRDXs protein family, PRDX1 is considered an important antioxidant protein, can regulate gene expression, and also enhances the killing activity of NK cells against cancer cells^38^, thereby preventing malignant transformation of cells. It has been shown that decreased PRDX1 levels lead to impaired antioxidant response and excessive accumulation of ROS, promoting hepatocellular carcinoma cell death^39^. In the present study, we found that PRDX1 expression levels were significantly upregulated in hepatocellular carcinoma cell lines, especially in HEPG2 cells. Furthermore, high PRDX1 expression was associated with poor prognosis of hepatocellular carcinoma, and silencing PRDX1 increased the accumulation of Fe2+ and led to lipid peroxidation accumulation in hepatocellular carcinoma cells, which promoted ferroptosis in hepatocellular carcinoma. Therefore, PRDX1 can be used as a potential biomarker for the prognostic value of hepatocellular carcinoma and can serve as a therapeutic target for hepatocellular carcinoma.

In summary, we established a prognostic model with four ferroptosis-related genes through statistical analyses and processes another time in the testing cohort to evaluate its accuracy. Meanwhile, we also checked the relevance between the overall survival rate and the expression level of each prognostic gene, respectively. And we selected PRDX1 as the key which silencing can promoted ferroptosis in hepatocellular carcinoma according to the experimental validation. In addition, we check the functions and pathways of genes to validate the connection between genes and ferroptosis. It can also demonstrate that the ferroptosis-related gene signature can be a predictor in HCC, linking ferroptosis and HCC more closely. However, the limitation in this study needs improvement. The datasets we acquired both are from public databases, the quantity of samples and the completeness of clinical information need to collect more to strengthen its reliability. And we also need a forward-looking study to further improve the availability of the prognostic model.

## Methods

### Data acquisition

Expression RNA-seq (FPKM value), simple nucleotide variation data, and clinical information of patients were obtained from the Cancer Genome Atlas (TCGA) (https://portal.gdc.cancer.gov/repository) dataset as a training cohort. The validation cohort was downloaded from the ICGC portal (https://dcc.icgc.org/projects/LIRI-JP). A list of 214 ferroptosis-related genes (FRGs) was acquired from the FerrDb database (http://www.zhounan.org/ferrdb), a web-based database of ferroptosis regulators and markers and ferroptosis-disease associations^40^.

### Differential expression analysis

To identify differentially expressed genes (DEGs) between tumor tissues and normal tissues, we used the “limma” package in R (version 4.0.2) to calculate the logFC (log fold change) and *P* values of 214 ferroptosis-related genes. The DEGs were filtered using adjusted p-value (adjusted by false discovery rate) < 0.05 and absolute logFC > 0.5. Following this, the DEGs were separated into two groups (high-expressed and low-expressed groups) for simple nucleotide variation analysis. The “maftools” R package was used to analyze the summary of DEGs mutation information in the TCGA dataset. Besides, we utilized the Search Tool for Recurring Instances of Neighbouring Genes (STRING) database (https://string-db.org/) to analyze a PPI network of DEGs, and then the PPI was visualized by Cytoscape software (version 3.8.2).

### Identification of prognostic FRGs and signature building

A univariate Cox regression analysis was used to screen differentially expressed FRGs associated with overall survival (OS) in HCC patients, and we considered *P* value < 0.05 as statistical significance. To narrow the range of potential prognostic FRGs, we did a LASSO regression analysis by applying the “glmnet” package in R. Then, for performing the prognostic signature, we conducted the stepwise multivariate Cox regression analysis and constructed a ferroptosis-related four-gene signature. The prognostic risk score was calculated according to the expression levels of the genes and a linear combination of the regression coefficient (λ) in a multivariate Cox regression model. The formula was established as follows: score = sum (each gene’s expression level × λ). The patients were divided into a high-risk group and a low-risk group based on the cut-off value of the risk scores.

### Prognosis analysis

Using “survival”, “survminer” and “timeROC” packages in R software to plot Kaplan–Meier survival curves and ROC curves, which can evaluate the potentially predictive performances of the prognostic signature. GEPIA (Gene Expression Profiling Interactive Analysis) website (http://gepia.cancer-pku.cn/) also can analyze the expression level of genes in normal and tumor groups based on TCGA database and GTEx Portal (https://www.gtexportal.org/home/) and plot Kaplan–Meier survival curves of the selected gene. Finally, we conducted univariate and multivariate Cox regression analysis to confirm which traditional clinical characteristic was independent. Hazard ratios (HRs) and 95% confidence intervals (CIs) for each variable were calculated. *P* value < 0.05 was considered statistical significance.

### Immune cells and functions

Cell-type Identification By Estimating Relative Subsets Of RNA Transcripts (CIBERSORT) analysis was used to evaluate the proportion of 19 human immune cell subpopulations by calculating the absolute abundance of immune cells and stromal cells. *P* value < 0.05 indicates statistical significance. Making single-sample GSEA (ssGSEA) to estimate the immune scores of immune cells and abundance of immune functions in two risk groups by using the “GSVA” R package. Mann–Whitney test with *P* value validated its differential expression between the high-risk group and low-risk group.

### Functional enrichment analysis

To explore the functional annotation which was associated with the risk score, we conducted Gene Ontology (GO) and Kyoto Encyclopedia of Genes and Genomes (KEGG)^41–43^ analyses based on the DEGs (FDR < 0.05) by applying the “clusterProfiler” package^44^ in R software. P-values were adjusted with the FDR method. Next, we constructed the ferroptosis potential index (FPI) by using ssGSEA to reveal the functional roles of ferroptosis and differential FPI expression in tumor and normal tissues. Finally, we obtained the immunohistochemistry images of prognostic signature from the HPA website (The Human Protein Atlas, https://www.proteinatlas.org/).

### Cell culture and transfection

Human hepatocellular carcinoma cell lines (MHCC-97H, HEPG2, Huh-7) and human normal hepatocytes (LX2) were purchased from the American Type Culture Collection (ATCC). All cells were maintained in DMEM medium (Gibco, Grand Island, USA) containing 10% fetal bovine serum (Gibco, Grand Island, USA) and 1% penicillin–streptomycin (Gibco, Grand Island, USA) and incubated at 37 °C and 5% CO2. PRDX1- siRNA was obtained from Sangon Biotech, Shanghai, China (Shanghai, China) for silencing the expression of PRDX1. In this study, the PRDX1-siRNA sequence was: sense:5′- GCACCAUUGCUCAGGAUUATT -3′; antisense:5′- UAAUCCUGAGCAAUGGUGCTT -3′. Cells were seeded at a density of 6 × 104 cells/well in 12-well plates and transfected after 24 h. PRDX1-siRNA/ NC-siRNA was transfected using siRNA transfection reagent (Polyplus, France) to a final concentration of 20 nM. Finally, the expression levels of target proteins in cells transfected for 72 h were analyzed. The successfully transfected cells will be used for subsequent experiments.

### Western Blot

Proteins were extracted from cells and cell lysates were prepared using RIPA lysate with PMSF (Solarbio, Beijing, China), and then protein quantification was performed using a BCA protein assay kit (Sangon Biotech, Shanghai, China). Proteins were then separated by 12% SDS-PAGE and transferred to nitrocellulose membranes. The membranes were blocked with 5% bovine serum albumin (BSA) for 1 h and then incubated with primary antibody overnight at 4 °C. The next day, after washing the membrane three times with TBST, the membrane was incubated for 1 h at room temperature with horseradish peroxidase-labelled secondary antibody (1:4000), followed by three washes with TBST. Finally, the colors were developed using BeyoECL Moon (Beyotime Biotechnology, Shanghai, China).

### Lipid peroxidation assay

C11-BODIPY 581/591 (10 µM; ABclonal, Wuhan, China) was added to transfected HEPG2 cells and incubated at 37 °C and 5% CO2 for 1 h. Cells were then washed twice with PBS, digested with trypsin, then resuspended using PBS containing 5% FBS, and finally analysed by flow cytometry.

### Iron assay

Cells were detected for ferrous (Fe2+) levels using FerroOrange (Dojindo, China). Cells were incubated with FerroOrange for 0.5 h according to the instructions. Finally, cells were observed under a fluorescence microscope (BioT ek Cytation 5, BioT ek, USA).

### Statistical analysis

Data are expressed as mean ± standard deviation (SD). Statistical analysis was performed by using GraphPad Prism analysis software. The *t* test was used to assess the difference between the two groups and a value of *P* < 0.05 indicates a statistically significant difference, * indicates *P* < 0.05; ** indicates *P* < 0.01; *** indicates *P* < 0.001.

## Supplementary Information

Supplementary Information 1.

Supplementary Information 2.

## Data Availability

The original contributions presented in the study are included in the article/supplementary material, further inquiries can be directed to the corresponding authors.
