# Supplementary material for: Identification of ferroptosis-related genes for overall survival prediction in hepatocellular carcinoma
Source: Sci Rep. 2022 Jun 15;12:10007. doi: 10.1038/s41598-022-14554-7 (PMC9200861; doi:10.1038/s41598-022-14554-7)

**Supplementary data**


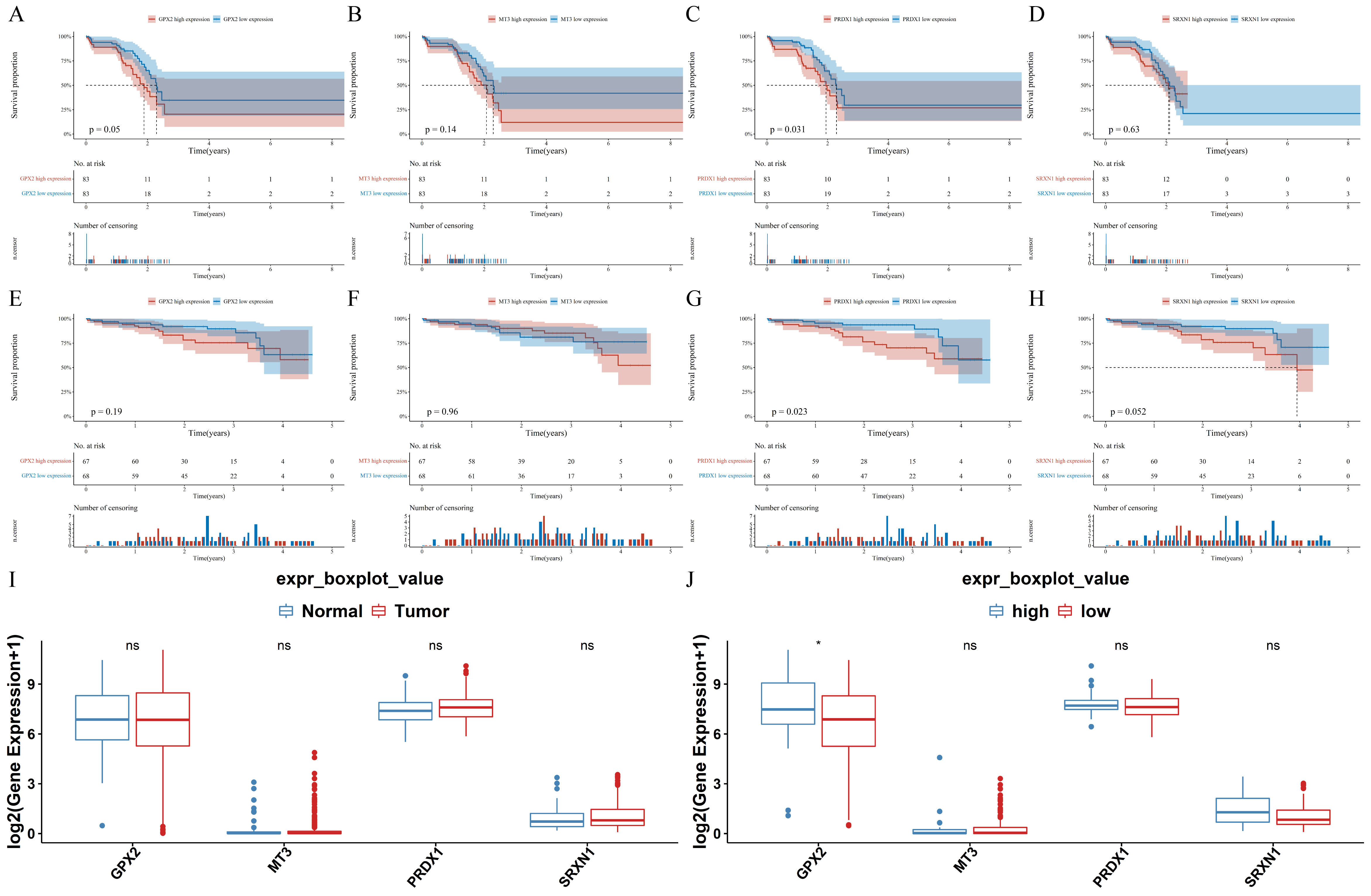


**Fig. S1.** The survival analysis and the difference of expression level in the normal, tumor, and risk groups of prognostic genes. (**A-H**) The survival analysis of four prognostic genes with their high and low expression level groups in the training and testing datasets, respectively. (**I**) The expression level of four prognostic genes in normal and tumor groups in the training cohort. *p=0.05; ns, not significant. (**J**) The expression level of four prognostic genes in the training cohort was measured in high- and low-risk groups. *p=0.05; ns, not significant.

**Fig. S2.** The process of screening key genes among prognostic genes. (**A, B**) The PPI of four prognostic genes with a minimum required interaction score whose are medium confidence (0.400) (**A**) and high confidence (0.700) (**B**), respectively. (**C**) The legend of **Fig. S2A, B**. (**D, E**) The expression level of PRDX1 (**D**) and SRXN1 (**E**) in normal and tumor groups. And red represents the tumor group but grey represents the normal group. *p=0.05. (**F, G**) The survival analysis of PRDX1 (**F**) and SRXN1 (**G**) with their high and low expression level groups, respectively.


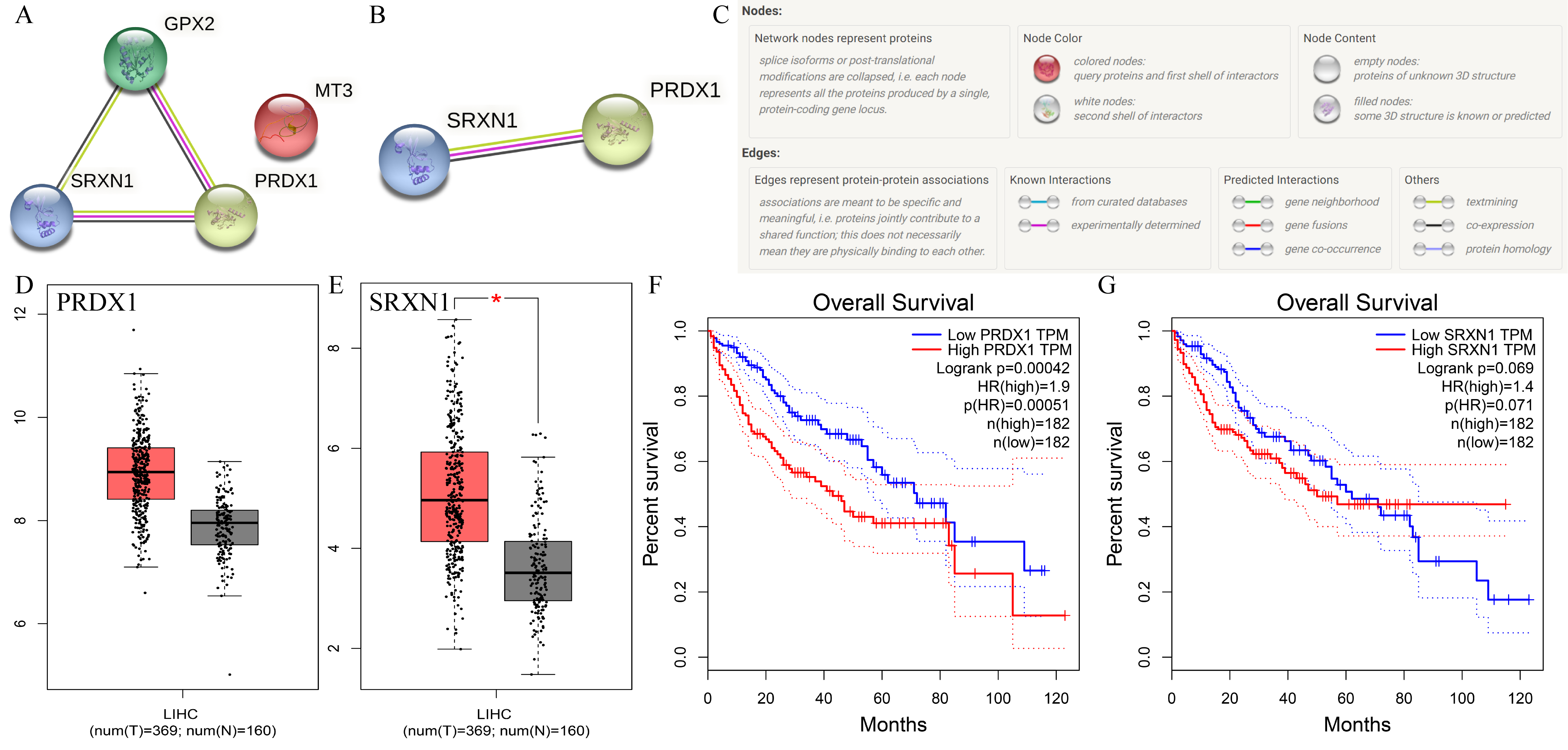

Supplement: Supplementary file 1 — Supplementary Information 1. [file 41598_2022_14554_MOESM1_ESM.docx]
