# Supplementary material for: Identification of ferroptosis-related genes for overall survival prediction in hepatocellular carcinoma
Source: Sci Rep. 2022 Jun 15;12:10007. doi: 10.1038/s41598-022-14554-7 (PMC9200861; doi:10.1038/s41598-022-14554-7)

The WB bands are tailored to the molecular weight of the target band before incubating the antibody and then incubating the target antibody.

Figure9.A

PRDX1

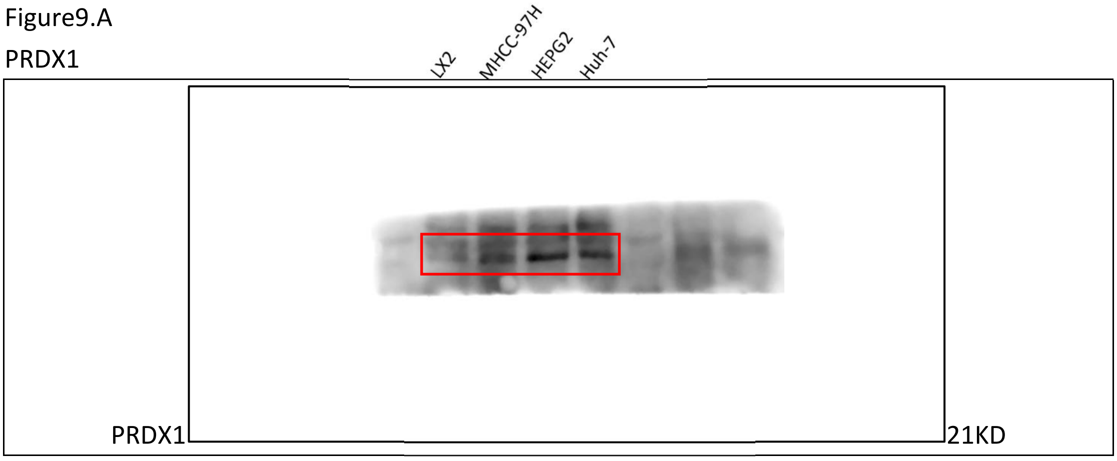

Figure9.A

GAPDH

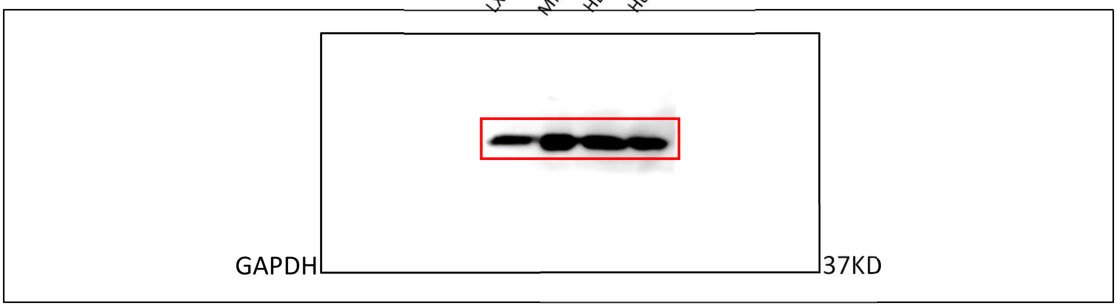

Figure9.B

PRDX1

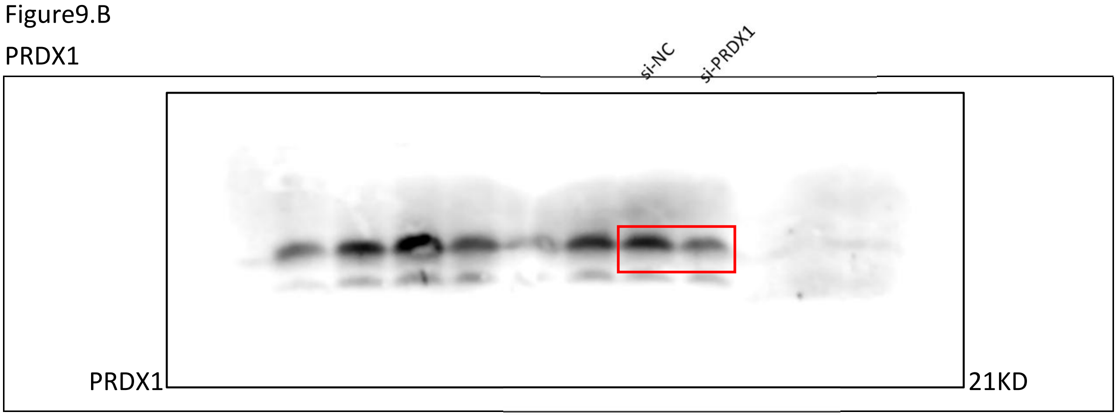

Figure9.E

SLC7A11

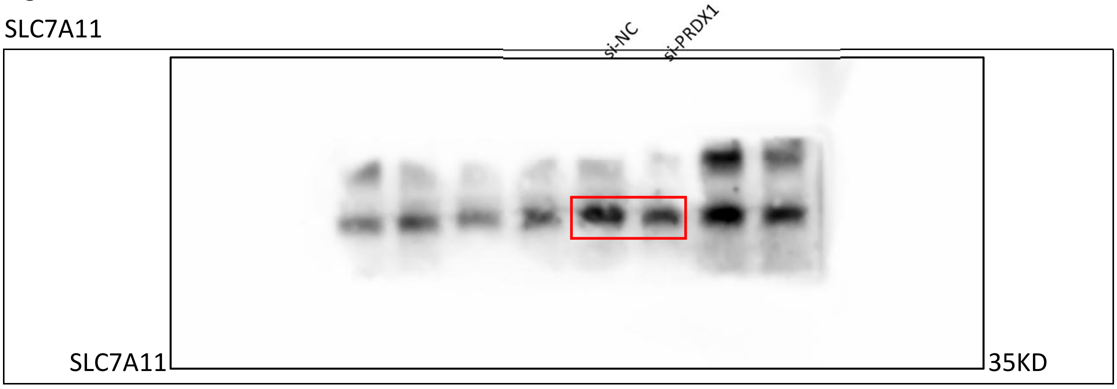

Figure9.E

ACSL4

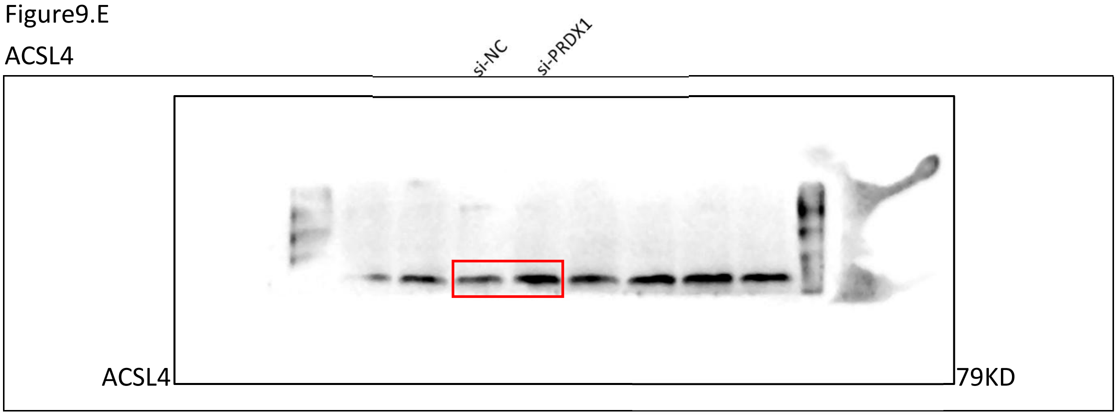

Figure9.E

GPX4

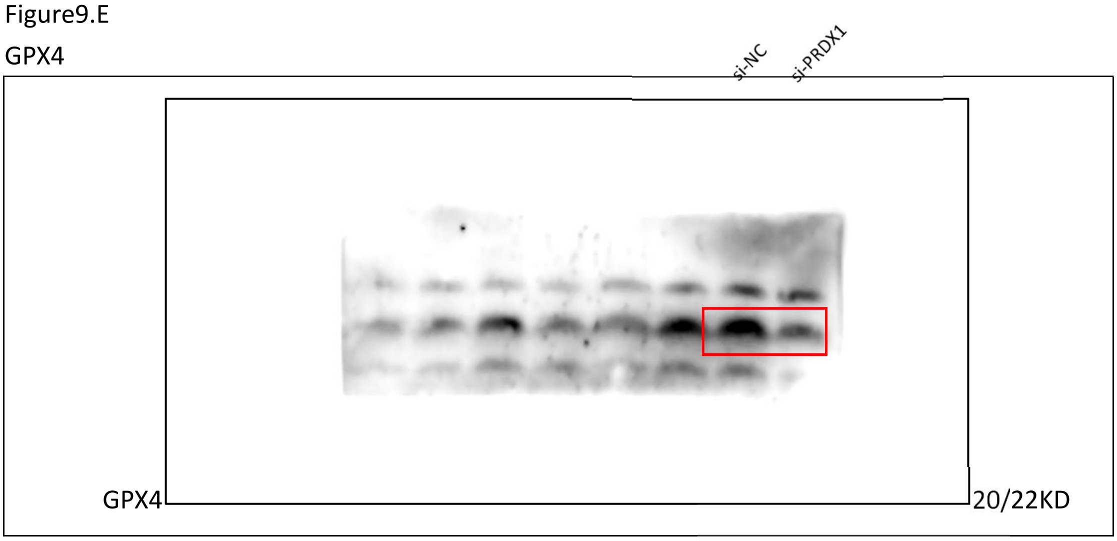

Figure9.E

4HNE

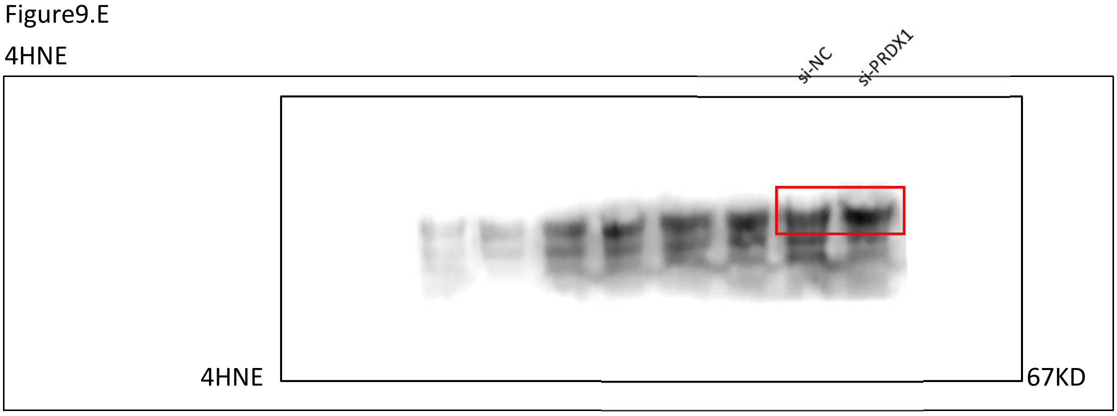

Figure9.B、E

GAPDH

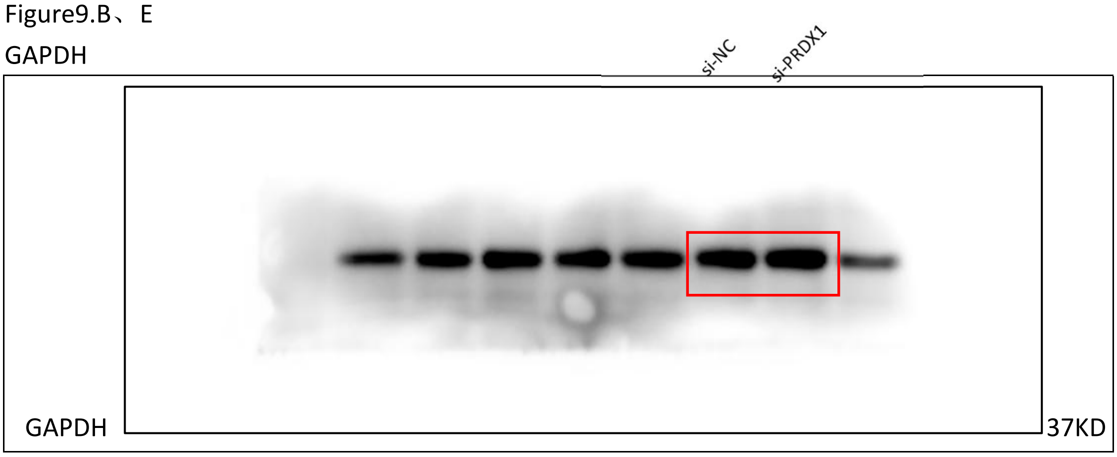

Supplement: Supplementary file 2 — Supplementary Information 2. [file 41598_2022_14554_MOESM2_ESM.pdf]
